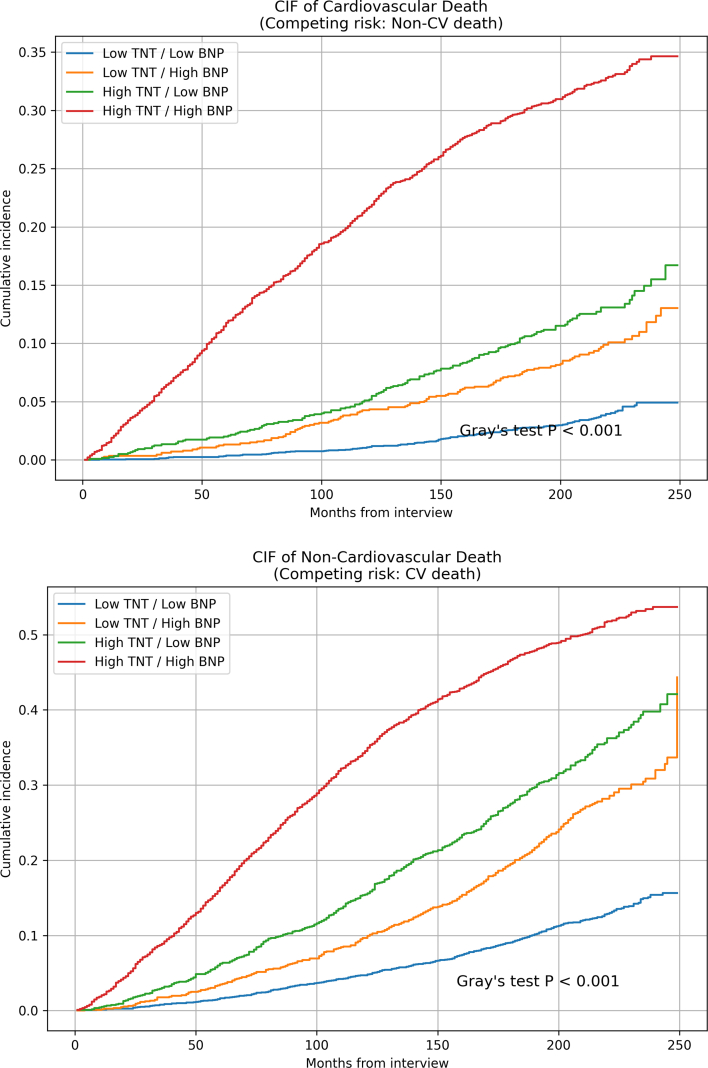# Toward practical screening of mortality risk: Insights from interpretable machine learning in NHANES^☆^

**DOI:** 10.1016/j.ijcrp.2026.200595

**Published:** 2026-02-12

**Authors:** Yi-Ting Lin, Lian-Yu Lin, Kai-Jen Chuang

**Affiliations:** a Department of Medicine, School of Medicine, College of Medicine, Taipei Medical University, Taipei, Taiwan; b Department of Internal Medicine, National Taiwan University Hospital, Taipei, Taiwan; c Department of Internal Medicine, College of Medicine, National Taiwan University, Taipei, Taiwan; d Department of Public Health, School of Medicine, College of Medicine, Taipei Medical University, Taipei, Taiwan; e School of Public Health, College of Public Health and Nutrition, Taipei Medical University, Taipei, Taiwan

**Keywords:** Mortality, Screening mortality, Machine learning, Troponin T, N-terminal pro B-Type natriuretic peptide, NHANES

## Abstract

**Background:**

Efficient community-based screening for individuals at high risk of mortality is a major public health challenge. While many predictors have been proposed, there is limited consensus on which factors are both robust and practical for population screening. This study applied interpretable machine learning to identify efficient predictors of all-cause and cardiovascular mortality in a nationally representative cohort.

**Methods:**

We analyzed 9957 adults aged ≥40 years from NHANES 1999–2004 with linked mortality follow-up. A total of 134 demographic, lifestyle, and biomarker variables were evaluated across multiple algorithms. Model interpretability was assessed with Shapley Additive Explanations (SHAP), and the prognostic implications of leading predictors were examined with Kaplan–Meier analyses.

**Results:**

Over 5 years, 1293 participants (13.0%) died. Across analytic approaches, age, troponin T (TNT), and N-terminal pro-B type natriuretic peptide (NT-proBNP) consistently emerged as the most influential predictors. Survival analyses demonstrated significantly poorer outcomes among individuals with elevated TNT and NT-proBNP. A parsimonious five-variable model (age, TNT, NT-proBNP, physical activity, gender) retained good discrimination (AUC = 0.841) and calibration.

**Conclusions:**

A parsimonious set of five predictors—age, gender, physical activity, TNT, and NT-proBNP—enabled efficient mortality risk stratification in NHANES, supporting their potential role in practical community screening.

## 1 Introduction

All-cause mortality is a central indicator of population health, and identifying individuals at heightened risk is critical for effective prevention strategies [1]. With aging populations and the growing burden of chronic diseases, there is increasing interest in community-based screening programs that can detect high-risk individuals early and enable timely intervention [2]. However, one of the persistent challenges in this field is to determine which screening approaches are both effective and cost-efficient for large-scale implementation.

Prior research has linked lifestyle behaviors, socioeconomic status, and comorbidities to mortality risk [3,4], and more recently, attention has turned to biological markers [[5], [6], [7]]. Yet, many studies rely on either traditional risk scores with limited predictive power or complex biomarker panels that are difficult to implement in routine practice. As a result, there remains a critical need to identify a parsimonious set of feasible predictors that balance predictive accuracy with practicality for use in community or population-level screening.

The National Health and Nutrition Examination Survey (NHANES), with its nationally representative sampling, comprehensive clinical and laboratory data, and linked mortality follow-up, offers a unique opportunity to address this gap. By leveraging this dataset, the present study aims to systematically evaluate a broad range of candidate variables and determine a concise set of predictors that can serve as the basis for efficient and scalable mortality risk screening strategies.

## 2 Methods

### 2.1 Data source and study population

We used data from the National Health and Nutrition Examination Survey (NHANES) 1999–2004, a nationally representative survey of the U.S. population with linked mortality follow-up [8]. A total of 31,126 participants were initially enrolled. For the present study, we restricted the cohort to individuals aged 40 years or older, resulting in 9977 eligible participants. After excluding 20 subjects without mortality follow-up information, 9957 individuals remained for the final analyses, contributing data on 134 demographic, clinical, laboratory, and lifestyle variables.

We conducted design-based sensitivity analyses incorporating examination weights, strata (SDMVSTRA), and primary sampling units (SDMVPSU). Survey-weighted analyses were applied to baseline characteristics and calibration assessments (including calibration curves, calibration-in-the-large, calibration slope, calibration intercept, and Brier score), while discrimination and SHAP-based feature ranking were reported from unweighted models. Our study was conducted using Python 3.13.7 along with compatible open-source packages for data analysis and ML model building.

### 2.2 Outcomes

The primary endpoint was all-cause mortality within 5 years of baseline interview. Secondary endpoints included cardiovascular mortality and non-cardiovascular mortality, classified using the NHANES-linked National Death Index.

### 2.3 Data preprocessing

Categorical variables were treated as nominal and encoded using one-hot encoding rather than ordinal coding to avoid imposing artificial ordering and spurious effects from assigning arbitrary values to unseen categories. Encoding was performed within each cross-validation training fold using handle_unknown = "ignore", so that categories not observed in the training split did not bias validation predictions.

Missing values were handled in a fold-specific manner to avoid information leakage. For XGBoost, continuous variables were left as NaN and handled internally by the model's optimal split-direction learning. For the remaining algorithms, continuous variables were imputed within each training fold using iterative multivariable imputation, while categorical variables were imputed with the most frequent category prior to one-hot encoding. Missing data patterns are summarized in Supplementary Table S1.

No discretization or categorization of continuous laboratory measures was applied during model training. No feature scaling/standardization was applied for tree-based models.

### 2.4 Parameters description

A total of 134 variables were analyzed, including basic characteristics (age, gender, race/ethnicity, annual household income), anthropometric and body composition measures (body mass index [BMI], circumferences of waist, arm, calf, and thigh, triceps and subscapular skinfolds, extracellular and intracellular fluid, and fat mass), cardiovascular fitness (estimated maximal oxygen consumption and fitness level), and comorbidities (hypertension, hyperlipidemia, diabetes mellitus, heart failure, myocardial infarction, and stroke). Laboratory examinations comprised complete blood count, blood biochemistry, serum cardiac markers (troponin T [TNT], N-terminal pro-B-type natriuretic peptide [NT-proBNP]), serum cotinine, insulin, C-peptide, C-reactive protein [CRP], renal function markers (β2-microglobulin [B2M], cystatin C, estimated glomerular filtration rate [eGFR]), thyroid hormones (thyroid stimulating hormone, thyroxine), sex hormones (testosterone, estradiol, sex hormone-binding globulin, androstenedione glucuronide), serum heavy metals (cadmium, lead, mercury), DNA methylation profiles (30 arrays), telomere length (mean and standard deviation), serum vitamin B12, and red blood cell folate. Physical activity was assessed by questionnaire (PAQ), categorized into four levels from sedentary to heavy work, with metabolic equivalent (MET) scores derived to quantify activity intensity.

### 2.5 Machine learning models and comparison

We compared four algorithms: logistic regression (GLM), random forests (RF), Extreme Gradient Boosting (XGBoost), and LightGBM. Model performance was assessed with 5-fold stratified cross-validation, generating out-of-fold predictions. Discrimination was quantified by the area under the receiver operating characteristic curve (ROC-AUC). Calibration was evaluated using the Brier score and logistic recalibration slope/intercept. Given the imbalanced event rate (13%), models were compared at two clinically prespecified operating points—PPV fixed at 40% and sensitivity fixed at 85%.

Calibration plots were constructed using quantile binning (10 bins), and ROC curves were generated from out-of-fold predictions. Models were compared using ROC-AUC as the primary metric, with the Brier score used as a complementary calibration metric (secondary criterion when differences were ≤0.005).

### 2.6 Model explainability with SHAP

For the best-performing models, feature contributions were examined using Shapley Additive explanations (SHAP) [9]. To avoid information leakage when SHAP was used to inform feature reduction, SHAP computation and ranking were performed within the development (training) data only, using 5-fold cross-validation: in each fold, SHAP global importance was defined as the mean absolute SHAP value (mean |SHAP|) computed on the fold-specific training data (subsampling up to 2000 observations for computational efficiency). SHAP interaction and dependence plots were used for interpretability and were computed using training-fold data only.

To investigate relationships among influential predictors, we summarized SHAP global importance across cross-validation training folds. The top ten features were then selected for correlation and clustering analyses.

Pairwise correlations were estimated using Spearman's rank coefficients to capture potential non-linear monotonic relationships. A distance matrix (1−|ρ|) was constructed and hierarchical agglomerative clustering with average linkage was applied, with the dendrogram and clustered heatmap used to identify groups of closely related predictors.

SHAP interaction values from TreeExplainer were used to quantify interactions in the XGBoost model, focusing on the TNT–NT-proBNP pair from the training set. SHAP dependence plots further visualized how the effect of TNT on model output varied across NT-proBNP levels.

### 2.7 Kaplan–Meier survival analysis

To assess prognostic relevance, Kaplan–Meier analyses were conducted within a nested cross-validation framework. In each outer fold, optimal cutoffs for key biomarkers (e.g., TNT and NT-proBNP) were determined in the inner training folds using the Youden index, and participants in the outer-fold held-out split were stratified into four groups (low/low, low/high, high/low, high/high). Survival curves were compared using the log-rank test to evaluate the consistency of risk stratification across outcomes.

Competing-risk analyses were further performed using Aalen–Johansen cumulative incidence functions with cardiovascular and non-cardiovascular death as competing events, applying median fold-specific cutoffs and comparing groups with Gray's test.

### 2.8 Final parsimonious model

We defined a five-variable parsimonious model using age, TNT, NT-proBNP, PAQ, and Gender. Candidate variables were first screened using SHAP-based importance derived only from the development data (training folds), and we examined the stability of variable rankings across clinically relevant subgroups. The final feature set was determined by jointly considering (i) consistent importance signals across subgroups, (ii) real-world data availability and feasibility for implementation, and (iii) parsimony. SHAP was used to inform and summarize these patterns rather than as the sole automated selection rule. PAQ and gender were treated as nominal variables. The dataset was randomly split into training (70%) and test (30%), with a stratified validation subset (15% of the training pool) used for early stopping and calibration. Class imbalance was addressed using the scale_ pos_weight parameter. An XGBoost classifier was trained using early stopping based on validation performance. Model discrimination was evaluated on the test set using ROC-AUC and PR-AUC.

Calibration was first quantified using internal cross-validation by estimating the calibration slope and calibration-in-the-large (intercept) of the raw model probabilities. Isotonic regression was then fitted on the validation set and applied to the test set to obtain recalibrated probabilities. On the test set, calibration performance was assessed using the Brier score before and after calibration, as well as the calibration slope and intercept (calibration-in-the-large) of the recalibrated predictions.

To provide clinically interpretable operating characteristics under class imbalance, performance at pre-specified clinically meaningful thresholds was reported on the calibrated test set, including a high-precision setting with positive predictive value (PPV) fixed at 40%, together with the corresponding sensitivity, specificity, and negative predictive value.

### 2.9 Temporal external validation

We used data from NHANES 1999–2002 cycles as the training cohort and NHANES 2003–2004 as an independent external validation cohort. To evaluate the incremental value of multimodal predictors beyond age and basic demographics, three parsimonious logistic regression models were constructed as benchmarks: Model A: Age only, Model B: Age and sex, Model C: Age and PAQ.

Model discrimination was assessed in the validation cohort using the area under the receiver operating characteristic curve (AUC), which is equivalent to the concordance index (C-index) for binary outcomes. The incremental predictive value of the main model over each benchmark model was quantified using the continuous net reclassification improvement (NRI) and the integrated discrimination improvement (IDI). NRI and IDI were computed in the validation cohort without predefined risk categories.

## 3 Results

### 3.1 Baseline characteristics

In the weighted cohort, individuals who died within 5 years were significantly older than survivors (69.4 ± 0.9 vs. 55.6 ± 0.3 years, p < 0.001) and were more likely to be male (57.9% vs. 46.6%, p < 0.001). The distribution of race/ethnicity differed between groups (p < 0.001), with a higher proportion of non-Hispanic Black participants and a lower proportion of Mexican Americans among decedents (Table 1).

**Table 1:** Baseline characteristics of the study population.

	Survived	Dead	p-value
N(weighted)	1.09E+08	7297538	
**Basic demographics**			
Age, years	55.63 ± 0.25	69.39 ± 0.86	<0.001
Male gender, %	46.6%	57.9%	<0.001
Race, %			<0.001
Mexican American	4.7	3.1	
Other Hispanic	4.3	4.1	
Non-Hispanic White	77.2	73.4	
Non-Hispanic Black	9.1	13.7	
Others	4.6	5.8	
**Risk factor**			
History of hypertension, %	36.6	49.9	<0.001
History of diabetes, %	9.7	18.5	<0.001
History of hyperlipidemia, %	20.7	20.7	0.977
**Comorbidity**			
Heart failure, %	2.6	12.2	<0.001
Heart attack, %	8.3	22.3	<0.001
Stroke, %	3.4	11.2	<0.001
**Laboratory**			
HbA1c, %	5.60 ± 0.02	5.79 ± 0.07	<0.001
HDL, mg/dL	52.84 ± 0.34	55.03 ± 1.3	0.0332
Total Cholesterol, mg/dL	209.87 ± 0.89	206.51 ± 2.79	0.0174
Glucose, mg/dL	100.84 ± 0.61	108.35 ± 2.89	0.077
Triglycerides, mg/dL	151.07 ± 3.57	149.40 ± 7.46	0.9431
eGFR, mL/min/1.73 m^2^ m^2^	88.54 ± 0.54	77.17 ± 2.11	<0.001

Abbreviations. HDL, high density lipoprotein; HbA1c, glycated hemoglobin; eGFR, estimated glomerular filtration rate.

Traditional cardiovascular risk factors were more prevalent in the deceased group, including hypertension (49.9% vs. 36.6%, p < 0.001) and diabetes (18.5% vs. 9.7%, p < 0.001), whereas the prevalence of hyperlipidemia was similar between groups. A history of cardiovascular disease was markedly more common among those who died, with substantially higher rates of heart failure (12.2% vs. 2.6%), myocardial infarction (22.3% vs. 8.3%), and stroke (11.2% vs. 3.4%) (all p < 0.001) (Table 1).

Laboratory profiles also differed between groups. Deceased participants had higher HbA1c levels (5.79 ± 0.07% vs. 5.60 ± 0.02%, p < 0.001) and fasting glucose, as well as modest differences in lipid measures(Table 1).

### 3.2 Model comparison

**Fig S1:**
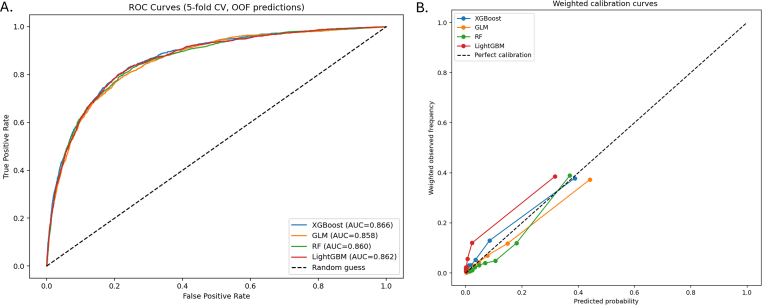
Model performance comparison across four algorithms. (A) Receiver operating characteristic (ROC) curves (B) Calibration curves comparing predicted and observed risk.

XGBoost demonstrated the best overall discrimination–calibration balance, achieving the highest ROC-AUC (0.866) and the lowest Brier scores, followed closely by LightGBM, random forests, and logistic regression (Table 2; Suppl. Fig. S1). Calibration analysis showed reasonable agreement between predicted and observed risk across models, with XGBoost and random forests exhibiting more favorable calibration slopes and lower overall prediction error, whereas LightGBM displayed a more compressed risk distribution(Table 2).To address class imbalance, models were compared at two prespecified operating points. At PPV = 40%, tree-based models showed much higher sensitivity than logistic regression, led by LightGBM (73.8%), followed by random forests (71.9%) and XGBoost(67.6%). LightGBM demonstrated more favorable clinical performance, but XGBoost was chosen for subsequent SHAP and Kaplan–Meier analyses because of its superior overall discrimination-calibration balance and more stable probability calibration for downstream interpretation.(Table 2).

**Table 2:** Model performance comparison across algorithms.

Model	XGBoost	LightGBM	RF	GLM
ROC-AUC	0.8660	0.8621	0.8597	0.8577
Brier(unweighted)	0.0818	0.0891	0.0834	0.0838
Brier(weighted)	0.0493	0.0529	0.0499	0.0504
Calibration slope(unweighted)	0.7732	0.5004	1.0627	0.8609
Calibration slope(weighted)	0.7804	0.5340	1.1597	0.8771
Calibration intercept(unweighted)	−0.0852	−0.0334	−0.0152	−0.1841
Calibration intercept(weighted)	−0.1444	−0.0301	−0.0244	−0.4340
PPV = 40%				
Sensitivity	0.6759	0.7378	0.7185	0.3596
Probability threshold	0.0572	0.0367	0.2100	0.2758
Observed PPV	0.4000	0.4000	0.4001	0.4002
Sensitivity = 85%				
PPV	0.2750	0.3075	0.3010	0.2273
Probability threshold	0.0074	0.0088	0.1200	0.1129
Observed sensitivity	0.8507	0.8507	0.8507	0.8507
Threshold = 0.5				
Precision	0.6332	0.6356	0.6571	0.6148
Recall	0.3658	0.3318	0.2475	0.3148
F1	0.4637	0.4360	0.3596	0.4165

### 3.3 Feature importance and explainability

**Fig. 1:**
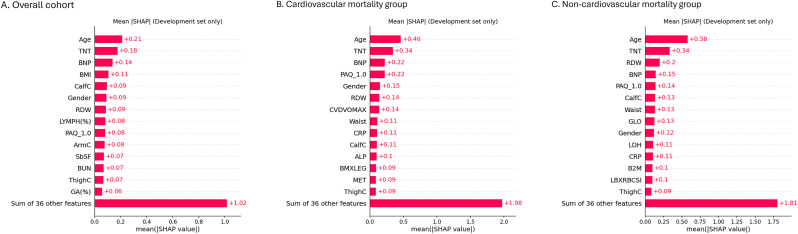
Global SHAP feature importance for all-cause and cause-specific mortality. (NT-proBNP, hereafter denoted as BNP in figures for simplicity).

Across all three subgroups, age and TNT consistently showed the largest global contributions to mortality risk (Fig. 1), ranking as the top two most important features in all groups. NT-proBNP was also among the leading predictors in the overall cohort and cardiovascular mortality models.

**Fig. 2:**
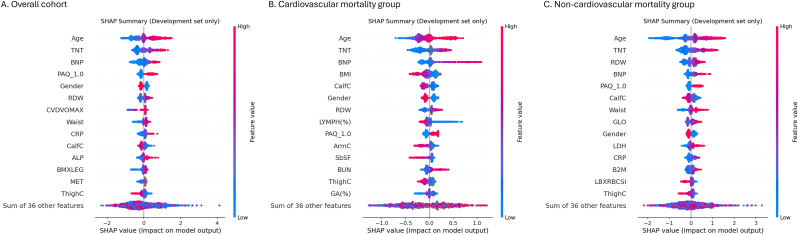
SHAP beeswarm plots showing direction and distribution of feature contributions.

**Fig. 3:**
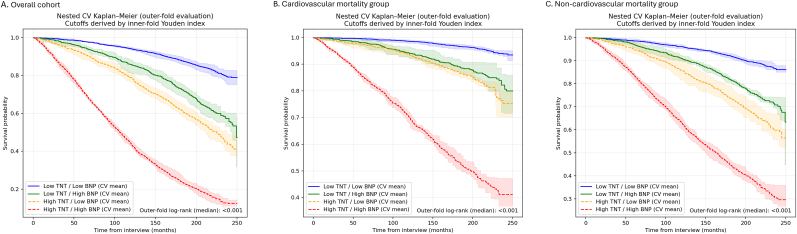
Kaplan–Meier survival curves stratified by TNT and NT-proBNP levels.

Directionality from the beeswarm plots (Fig. 2) demonstrated that higher age, TNT, and NT-proBNP values were consistently associated with increased mortality risk (red points distributed toward the positive SHAP side). Measures of body composition and nutritional status showed protective associations: larger thigh/calf circumference (ThighC/CalfC) and greater triceps skinfold tended to have negative SHAP values, whereas lower lymphocyte percentage (LYMPH%) and higher red-cell distribution width (RDW) were linked to higher risk. Greater physical activity level (high PAQ level) generally exerted a protective influence (see Fig. 3).

**Fig S2:**
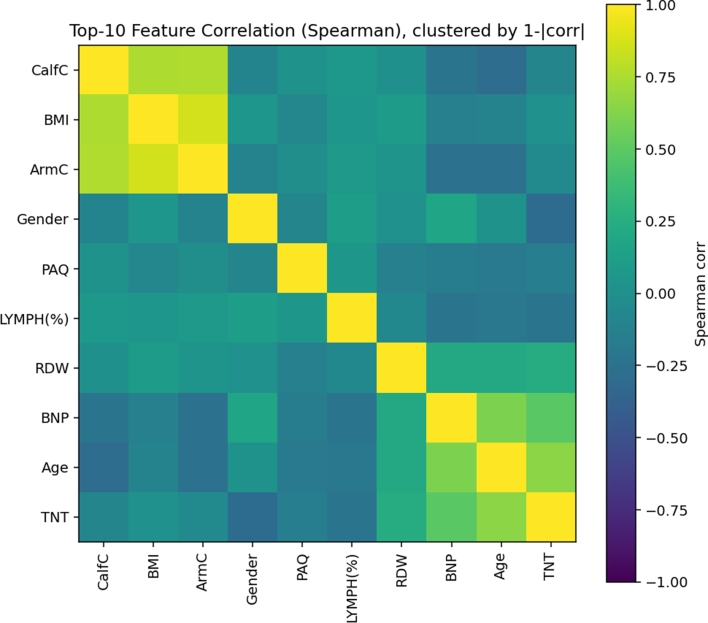
Correlation structure and hierarchical clustering of the top ten SHAP-ranked predictors.

Correlation and clustering of the top ten SHAP features identified three modules: a cardiac injury–stress axis (TNT, NT-proBNP, age, RDW), a body-composition cluster (CalfC, ArmC, BMI), and a demographic/immune–activity module (Gender, LYMPH(%), PAQ), with a moderate correlation between TNT and NT-proBNP (Spearman ρ ≈ 0.5–0.6; Suppl. Fig. S2).

**Fig S3:**
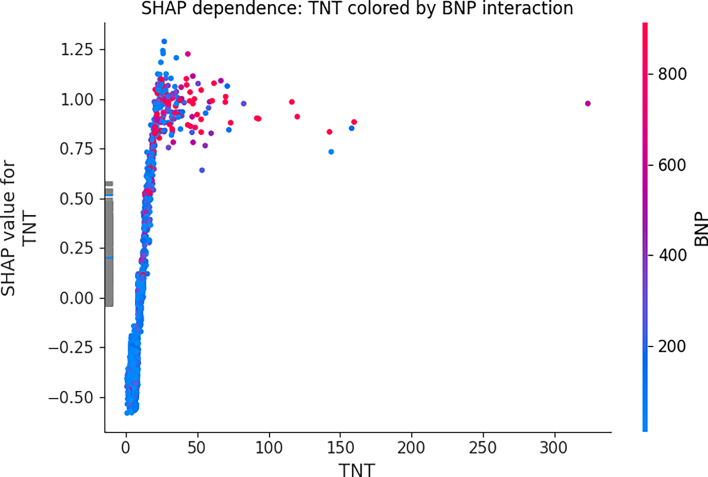
SHAP dependence plot illustrating the interaction between TNT and NT-proBNP in the training set.

SHAP interaction analysis showed negligible interaction between TNT and NT-proBNP, with interaction values near zero (Suppl. Table 2) and dependence plots demonstrating a monotonic effect of TNT that did not vary across NT-proBNP levels (Suppl. Fig. S3), supporting an approximately additive rather than synergistic contribution to risk prediction.

### 3.4 Kaplan–Meier survival analysis

Using nested cross-validation with inner-fold Youden index optimization, optimal cut-off values for TNT and BNP were derived (Suppl. Table S3) and used to stratify participants into four groups. Kaplan–Meier curves based on outer-fold held-out splits showed a clear, graded separation of survival across groups (Fig. 3).

In the overall cohort, participants with low TNT and low NT-proBNP had the best survival, whereas those with concomitantly high TNT and high NT-proBNP had the worst prognosis, with the two discordant groups showing intermediate risk (median outer-fold log-rank p < 0.001). The same hierarchical pattern was consistently observed for cardiovascular mortality and non-cardiovascular mortality (both median outer-fold log-rank p < 0.001), indicating additive prognostic value of TNT and NT-proBNP across different causes of death (Fig. 3).

**Fig S4:**
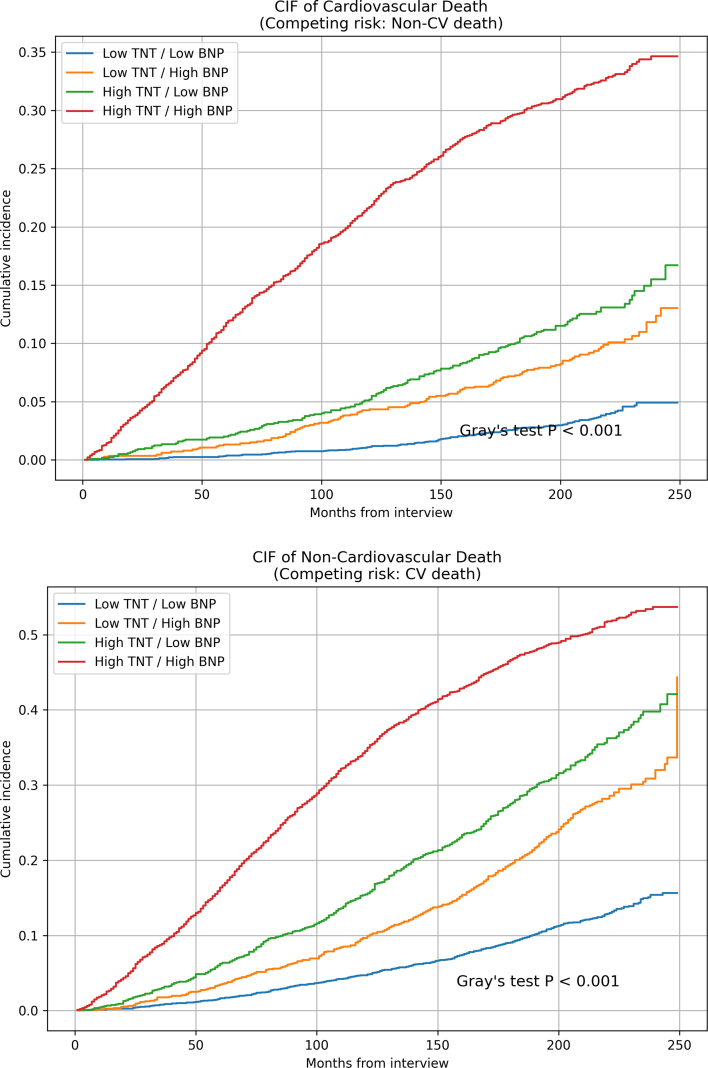
Cumulative incidence functions (CIFs) of cardiovascular death (upper panel) and non-cardiovascular death (lower panel). For the upper panel, non-cardiovascular death was treated as a competing event, whereas for the lower panel, cardiovascular death was treated as a competing event.

CIF analyses accounting for competing causes of death demonstrated consistent risk stratification for cardiovascular mortality across TNT/NT-proBNP groups, whereas group differences were less marked for non-cardiovascular death (Suppl. Fig. S4).

### 3.5 Final parsimonious model

**Fig. 4:**
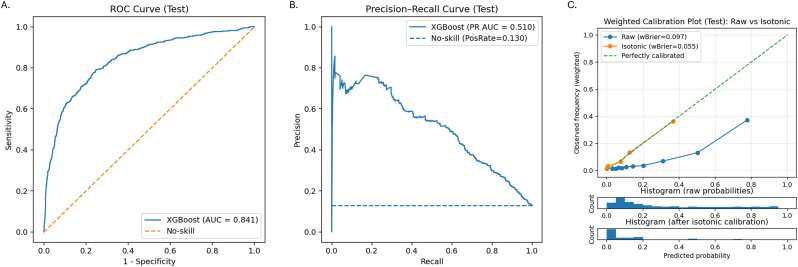
Performance of the final parsimonious XGBoost model including age, TNT, NT-proBNP, PAQ, and gender. (A) ROC curve in the held-out test split (AUC = 0.841). (B) Precision–recall curve (PR-AUC = 0.510). (C) Calibration plot before and after isotonic calibration.

A parsimonious XGBoost model incorporating five key predictors (age, TNT, NT-proBNP, PAQ, and gender) was developed. On the held-out test split, the model showed good discrimination with a ROC-AUC of 0.841 and a PR-AUC of 0.510, substantially exceeding the no-skill baseline defined by the event prevalence (∼13%) (Fig. 4A and B).

Calibration of the raw model probabilities was first examined using internal cross-validation. The cross-validated calibration slope was 0.84 and the calibration-in-the-large (intercept) was −1.69 (survey-weighted), indicating systematic over-confidence and global risk overestimation of the original XGBoost scores. Similar miscalibration was observed on the held-out test split before recalibration, with a weighted calibration slope of 0.90 and an intercept of −1.81 (Suppl. Table S4).

To address miscalibration, isotonic regression was fitted on the validation set and applied to the test set, markedly improving absolute risk accuracy (survey-weighted Brier score: 0.097 to 0.055; Fig. 4C). After recalibration, the weighted intercept shifted toward zero (−0.73), indicating correction of systematic risk overestimation, while the calibration slope decreased to 0.48, reflecting the expected compression of risk dispersion with nonparametric monotonic adjustment (Suppl. Table S5).

At a pre-specified high-precision operating point (PPV = 40%), the corresponding threshold was 0.44, yielding a sensitivity of 0.32, specificity of 0.98, and negative predictive value of 0.95, with 4.6% of participants classified as high risk (Suppl. Table S6).

### 3.6 Temporal external validation

In the external validation cohort (NHANES 2003–2004), the multimarker main model (Age, Gender, PAQ, TNT, and BNP) showed superior discrimination with an AUC of 0.853, outperforming the age-only (0.802), age-plus-gender (0.812), and age-plus-PAQ (0.817) benchmark models. Calibration of the main model was acceptable, with a slope of 0.96, indicating minimal overfitting, although a negative calibration intercept (CITL = −1.74) suggested modest overall risk overestimation (Suppl. Table S7).

Reclassification analyses demonstrated significant incremental value of the main model, with continuous NRI and IDI of 0.095 and 0.236, respectively, compared with the age-only model. Similar improvements were observed when compared with the age-plus-gender (NRI = 0.069; IDI = 0.224) and age-plus-PAQ (NRI = 0.062; IDI = 0.205) models, indicating enhanced risk stratification by incorporating cardiac biomarkers and physical activity into the prediction model(Suppl. Table S7).

## 4 Discussion

Community-based identification of individuals at high mortality risk remains a public health priority, yet existing approaches either rely on traditional risk scores with limited predictive power or on complex biomarker panels that are impractical for large-scale use [[10], [11], [12]]. Using the nationally representative NHANES cohort with long-term mortality follow-up, we systematically evaluated candidate predictors and identified a small set of readily obtainable markers—age, physical activity, and two circulating biomarkers—that consistently predicted all-cause and cardiovascular mortality. These results support the feasibility of parsimonious, cost-effective screening strategies for community and population-level risk stratification.

In this study, we used an interpretable ML framework to identify concise, actionable determinants of 5-year all-cause mortality. Recent large-scale studies and reviews strongly support our finding that elevated troponin levels are associated with increased all-cause mortality [[13], [14], [15]]. Particularly regarding the prediction of non-cardiovascular mortality risk, our study emphasizes the role of troponin. This observation is well supported by multiple population-based and clinical studies. In a large community-based investigation of nearly 20,000 individuals, Roos et al. demonstrated that higher concentrations of TNT were strongly and progressively associated not only with cardiovascular mortality but also with non-cardiovascular causes of death, including infections and chronic systemic conditions [16]. Moreover, in community-based cohorts such as ARIC, troponin elevations were associated with deaths from non-cardiovascular causes, particularly respiratory mortality [17]. Collectively, these data indicate that circulating troponin is not a marker limited to cardiovascular outcomes but a powerful integrative indicator of overall health status and vulnerability to non-cardiovascular causes of death.

Our results also highlight the complementary prognostic value of TNT and NT-proBNP. BNP and its inactive fragment NT-proBNP have long been established as robust predictors of heart failure and mortality. In seminal community-based work, Wang and colleagues demonstrated that even modest elevations of natriuretic peptides strongly predicted cardiovascular events and all-cause death in otherwise healthy populations [5]. Subsequent studies extended these observations to cohorts with comorbidities. In the ADVANCE trial, for example, both TNT and NT-proBNP were independently associated with cardiovascular outcomes and all-cause mortality among patients with type 2 diabetes, with their joint use offering superior risk discrimination [18]. Similarly, data from the MESA study confirmed that NT-proBNP and TNT each contributed incrementally to risk prediction, identifying distinct high-risk subgroups across glycemic strata [19]. Mechanistically, this complementarity is consistent with the notion that troponin elevations primarily reflect ongoing myocardial injury, whereas BNP elevation reflects hemodynamic overload. Comparative analyses across community cohorts, diabetic populations, and high-risk hypertensive patients support this additive effect, demonstrating that combined assessment of TNT and BNP more effectively stratifies outcomes than either biomarker alone.

This study has several limitations. Although we performed a temporal validation using later NHANES cycles (2003–2004), we did not conduct external validation in an entirely independent cohort outside NHANES. Therefore, the transportability of our parsimonious model to other populations and healthcare settings remains uncertain. A further limitation is that we examined only 5-year mortality, without adjudicated non-fatal cardiovascular events, and analyzed participants with and without prior cardiovascular disease together. While appropriate for population screening, this differs from clinical practice, which uses separate models for primary and secondary prevention. Future studies should develop and validate stratified models for fatal and non-fatal outcomes in these two groups.

## 5 Conclusion

In this nationally representative cohort, we demonstrated that a small set of accessible markers—age, gender, physical activity, TNT, and NT-proBNP—were the most influential predictors of all-cause mortality. Importantly, TNT showed strong prognostic value not only for cardiovascular but also for non-cardiovascular mortality, underscoring its role as systemic indicators of biological vulnerability. By integrating explainable machine learning with survival analysis, we highlighted both the predictive utility and the long-term prognostic implications of these factors. Future studies should validate these findings in contemporary cohorts, assess longitudinal biomarker dynamics, and explore integration with existing clinical risk scores.

**Table:** List of abbreviations

B2M	β2-Microglobulin
BNP	B-type Natriuretic Peptide
BP	Blood Pressure
BUN	Blood Urea Nitrogen
CalfC	Calf Circumference
CRP	C-Reactive Protein
eGFR	Estimated Glomerular Filtration Rate
GLM	Generalized Linear Model (Logistic Regression)
HbA1c	Hemoglobin A1c
HDL	High-Density Lipoprotein
KM	Kaplan–Meier
LDH	Lactate Dehydrogenase
LDL	Low-Density Lipoprotein
LYMPH%	Lymphocyte Percentage
MET	Metabolic Equivalent of Task
NT-proBNP	N-terminal pro B-type Natriuretic Peptide
PAQ	Physical Activity Questionnaire
RF	Random Forests
RDW	Red Cell Distribution Width
TG	Triglycerides
TNT	Troponin T
ThighC	Thigh Circumference

## CRediT authorship contribution statement

**Yi-Ting Lin:** Writing – original draft, Visualization, Software, Methodology, Formal analysis, Data curation. **Lian-Yu Lin:** Writing – review & editing, Methodology, Funding acquisition and Supervision. **Kai-Jen Chuang:** Writing – review & editing, Supervision, Conceptualization.

## Declarations of interest

None declared.

## Figures and Tables

**Figure:**