# Supplementary material for: Toward practical screening of mortality risk: Insights from interpretable machine learning in NHANES
Source: Int J Cardiol Cardiovasc Risk Prev. 2026 Feb 12;29:200595. doi: 10.1016/j.ijcrp.2026.200595 (PMC12969032; doi:10.1016/j.ijcrp.2026.200595)
Supplement: Multimedia component 1 [file mmc1.docx]

**Supplement**

**Table S1.** Missing data patterns and effective sample sizes

| Variable | Missing | Valid | Variable | Missing | Valid |
| --- | --- | --- | --- | --- | --- |
| Gender | 0 | 9957 | White blood cell count | 1226 | 8731 |
| Age | 0 | 9957 | Lymphocyte percentage | 1267 | 8690 |
| Race | 0 | 9957 | Monocyte percentage | 1267 | 8690 |
| Income | 1139 | 8818 | Neutrophil percentage | 1267 | 8690 |
| Resting heart rate | 1179 | 8778 | Eosinophil percentage | 1267 | 8690 |
| Extracellular fluid | 8176 | 1781 | Basophil percentage | 1267 | 8690 |
| Total water body | 8176 | 1781 | Lymphocyte count | 1267 | 8690 |
| Intracellular fluid | 8176 | 1781 | Monocyte count | 1267 | 8690 |
| Fat free mass | 8176 | 1781 | Neutrophil count | 1267 | 8690 |
| Fat mass | 8176 | 1781 | Eosinophil count | 1267 | 8690 |
| Body mass index | 1209 | 8748 | Basophil count | 1267 | 8690 |
| Upper leg length | 1395 | 8562 | Red blood cell count | 1226 | 8731 |
| Calf circumference | 1274 | 8683 | Hemoglobin | 1226 | 8731 |
| Upper arm length | 1218 | 8739 | Hematocrit | 1226 | 8731 |
| Arm circumference | 1217 | 8740 | Mean cell volume | 1226 | 8731 |
| Waist circumference | 1350 | 8607 | Mean cell hemoglobin | 1226 | 8731 |
| Thigh circumference | 1464 | 8493 | MCHC | 1226 | 8731 |
| Triceps skinfold | 1935 | 8022 | Red cell distribution width | 1226 | 8731 |
| Subscapular skinfold | 2965 | 6992 | Platelet count | 1226 | 8731 |
| Predicted VO2max | 8817 | 1140 | Plasma glucose | 7128 | 2829 |
| Heart rate before exercise | 9035 | 922 | C-peptide | 7167 | 2790 |
| Heart rate increment | 8934 | 1023 | Insulin | 7166 | 2791 |
| Maximum heart rate | 8934 | 1023 | C-reactive protein | 1350 | 8607 |
| Heart rate recovery 1min | 9072 | 885 | Bone alkaline phosphatase | 3588 | 6369 |
| Heart rate recovery 2 min | 9034 | 923 | Total cholesterol | 1406 | 8551 |
| Heart rate recovery 3min | 9533 | 424 | High density lipoprotein | 1408 | 8549 |
| Estimated VO2max | 9050 | 907 | Albumin | 1416 | 8541 |
| Cardiovascular fitness level | 9050 | 907 | ALT | 1416 | 8541 |
| Glycohemoglobin | 1238 | 8719 | AST | 1416 | 8541 |
| Phosphorus | 1416 | 8541 | Alkaline phosphatase | 1416 | 8541 |
| Bilirubin | 1417 | 8540 | BUN | 1416 | 8541 |
| Total protein | 1419 | 8538 | Calcium | 1416 | 8541 |
| Triglyceride | 1415 | 8542 | Total cholesterol | 1414 | 8543 |
| Uric acid | 1416 | 8541 | Gamma-GT | 1416 | 8541 |
| Sodium | 1416 | 8541 | Serum glucose | 1416 | 8541 |
| Potassium | 1416 | 8541 | Iron | 1417 | 8540 |
| Chloride | 1419 | 8538 | LDH | 1418 | 8539 |
| Osmolality | 1416 | 8541 | Telomere length | 4940 | 5017 |
| Globulin | 1419 | 8538 | SD of mean Telomere length | 4940 | 5017 |
| Physical activity | 5 | 9952 | Hypertension history | 0 | 9957 |
| MET score | 5216 | 4741 | 30 variables of DNA methylation | 7425 | 2532 |
| NT-proBNP | 1990 | 7967 | Beta 2 Microglobulin | 1944 | 8013 |
| Testosterone | 9356 | 601 | Cystatin | 1938 | 8019 |
| Sex hormone binding protein | 9359 | 598 | Glycated albumin | 1945 | 8012 |
| Estradiol | 9356 | 601 | eGFR | 1416 | 8541 |
| Androstanedione glucuronide | 9365 | 592 | Heart failure history | 0 | 9957 |
| Troponin-T | 1992 | 7965 | Coronary artery disease history | 0 | 9957 |
| Cadmium | 6963 | 2994 | Cerebrovascular event history | 0 | 9957 |
| Lead | 6963 | 2994 | Taking hyperlipidemia medication | 0 | 9957 |
| Folate(RBC) | 6970 | 2987 | Taking hypertension medication | 0 | 9957 |
| Mercury | 9603 | 354 | Taking diabetes medication | 0 | 9957 |
| Vitamin B12 | 7011 | 2946 | Diabetes history | 0 | 9957 |
| Cotinine | 4172 | 5785 |  |  |  |

**Table S2.** TNT and NT-proBNP interaction summary

| mean(\|interaction\|) | mean(interaction) | median(interaction) | 95% quantile(\|interaction\|) |
| --- | --- | --- | --- |
| 0 | 0 | 0 | 0 |

**Table S3.** Cut-off values for TNT and NT-proBNP

| **fold** | **n_train** | **n_test** | **TNT_cutoff** | **NT-proBNP_cutoff** |
| --- | --- | --- | --- | --- |
| 1 | 6356 | 1590 | 7.57 | 85.41 |
| 2 | 6357 | 1589 | 7.59 | 75.31 |
| 3 | 6357 | 1589 | 7.57 | 85.41 |
| 4 | 6357 | 1589 | 7.49 | 81.62 |
| 5 | 6357 | 1589 | 7.41 | 75.31 |

**Table S4.** Cross-validated calibration of the raw model

| **Setting** | **Slope** | **Intercept (CITL)** | **Weighting** |
| --- | --- | --- | --- |
| **CV (raw)** | 0.79 | −0.086 | Unweighted |
| **CV (raw)** | 0.84 | −1.69 | weighted |

**Table S5.** Test-set calibration before and after isotonic regression

| **Model** | **Brier (weighted)** | **Slope** | **Intercept (CITL)** |
| --- | --- | --- | --- |
| **Raw (test)** | 0.097 | 0.898 | −1.807 |
| **Isotonic (test)** | 0.055 | 0.479 | −0.725 |

**Table S6.** Clinically meaningful operating points (PPV fixed at 40%)

| **Setting (Test, weighted, isotonic)** | **Threshold** | **Sensitivity** | **Specificity** | **PPV** | **NPV** | **Predicted positive rate** |
| --- | --- | --- | --- | --- | --- | --- |
| **PPV fixed at 40%** | 0.44 | 0.316 | 0.976 | 0.40 | 0.947 | 0.046 |

**Table S7.** Discrimination, calibration, and reclassification performance of the main multimarker model and three benchmark models in the external validation cohort

| **Model** | **AUC (C-index)** | **CITL** | **Slope** | **Brier** | **NRI** | **IDI** |
| --- | --- | --- | --- | --- | --- | --- |
| **Model** | 0.853 | -1.74 | 0.96 | **0.149** |  | |
| **Model A (Age)** | 0.802 | -0.006 | 0.98 | 0.102 | **0.095** | **0.236** |
| **Model B (Age+Gender)** | 0.812 | 0.003 | 0.99 | 0.100 | **0.069** | **0.224** |
| **Model C (Age+PAQ)** | 0.817 | -0.008 | 0.96 | **0.098** | **0.062** | **0.205** |

**Supplementary Figure legends**

**Figure S1.** Model performance comparison across four algorithms.
(A) Receiver operating characteristic (ROC) curves
(B) Calibration curves comparing predicted and observed risk..

**Figure S2.** Correlation structure and hierarchical clustering of the top ten SHAP-ranked predictors.

**Figure S3.** SHAP dependence plot illustrating the interaction between TNT and NT-proBNP in the training set.

**Figure S4.** Cumulative incidence functions (CIFs) of cardiovascular death (upper panel) and non-cardiovascular death (lower panel). For the upper panel, non-cardiovascular death was treated as a competing event, whereas for the lower panel, cardiovascular death was treated as a competing event.
